# Thyroid Function in Pregnant Women With Moderate to Severe Alcohol Consumption Is Related to Infant Developmental Outcomes

**DOI:** 10.3389/fendo.2018.00294

**Published:** 2018-06-05

**Authors:** Kirsten A. Donald, Catherine J. Wedderburn, Whitney Barnett, Nadia Hoffman, Heather J. Zar, Eva E. Redei, Dan J. Stein

**Affiliations:** ^1^Division of Developmental Paediatrics, Department of Paediatrics and Child Health, Red Cross War Memorial Children’s Hospital, University of Cape Town, Cape Town, South Africa; ^2^Department of Clinical Research, London School of Hygiene & Tropical Medicine, London, United Kingdom; ^3^Unit on Child and Adolescent Health, South African Medical Research Council (SAMRC), Cape Town, South Africa; ^4^Department of Paediatrics and Child Health, Red Cross War Memorial Children’s Hospital, University of Cape Town, Cape Town, South Africa; ^5^Department of Psychiatry and Mental Health, University of Cape Town, Cape Town, South Africa; ^6^Department of Psychiatry and Behavioral Sciences, Feinberg School of Medicine, Northwestern University, Chicago, IL, United States; ^7^Department of Physiology, Feinberg School of Medicine, Northwestern University, Chicago, IL, United States; ^8^Unit on Risk and Resilience in Mental Disorders, South African Medical Research Council (SAMRC), Cape Town, South Africa

**Keywords:** prenatal thyroid function, prenatal alcohol exposure, fetal alcohol spectrum disorders, infant development, pregnancy thyroid, thyroid function, PAE, child-development

## Abstract

**Introduction:**

Fetal alcohol spectrum disorders (FASD) have an estimated global prevalence of 2–5% of births, but prevalence is reported to be as high as 15.5% for FASD in certain high-risk communities in South Africa. Preclinical studies demonstrate that alcohol consumption during pregnancy interferes with thyroid hormone availability and function and negatively impacts exposed offspring. Very little is currently reported on this phenomenon in humans.

**Methods:**

This pilot study was embedded in the Drakenstein Child Health Study, a multi-disciplinary longitudinal birth cohort study investigating the early biological and psychosocial determinants of child health in South Africa. Twenty one mothers and their children with moderate–severe prenatal alcohol exposure (PAE) and 19 mothers and their children with no alcohol exposure were investigated. Maternal exposure history and blood samples were collected in mid-pregnancy and analyzed for serum-free thyroxin (FT4), free triiodothyronine (FT3), and thyroid stimulating hormone (TSH). Children were assessed with formally measured growth parameters and development was evaluated using the Bayley III Scales of Infant and Toddler Development (BSID III) at 6 and 24 months of age.

**Results:**

While there were no significant differences in serum TSH and FT4 between groups, FT3 levels were significantly higher in mothers with moderate–severe prenatal alcohol use. In abstinent pregnant women, levels of FT4 were significantly correlated with infants’ scores on cognitive measures at 6 and 24 months of age and with levels of gross motor skills at 24 months. However, in mothers with alcohol use, FT4 levels were not correlated with any cognitive or motor skills, but FT3 levels were significantly associated with scores on children’s social-emotional development at 24 months of age.

**Discussion:**

Thyroid function in PAE is sufficiently disrupted to lead to alterations in serum FT3 levels. The contrast in findings between PAE and abstinent dyads in their association of maternal thyroid function and infant development further suggests that such disruption is present and may contribute to adverse neurodevelopment. Further work is needed to determine the relationship between peripheral thyroid indices during pregnancy and neurodevelopmental outcomes in the context of PAE.

## Introduction

The World Health Organization (1) states that harmful alcohol usage ranks in the top five global contributors to disease, disability, and mortality. Fetal alcohol syndrome (FAS), the most severe of all fetal alcohol spectrum disorders (FASD), is further recognized as one of the major disease and disability categories within alcohol-related disorders (2, 3). The global prevalence of fetal FASD is estimated at 2–5% of births (4–7), but in South Africa, prevalence is reported to be as high as 6.3% for FAS and 15.5% for FASD in certain high-risk communities (8).

Alcohol may directly harm the developing fetus, or act indirectly by, among other mechanisms, suppressing maternal and fetal thyroid function (9–11). There are a number of preclinical studies, which demonstrate that maternal alcohol consumption during pregnancy interferes with thyroid hormone availability or function (12–18). In humans, excessive alcohol consumption may decrease serum levels of triiodothyronine (T3) (19, 20), thyroxine (T4) (21–23), free T3 (FT3), and free T4 (FT4) (24, 25) and increase thyroid stimulating hormone (TSH) levels (26). Alcohol use during pregnancy has also been reported to lead to a significant suppression of newborn TSH levels (27, 28). One contrasting report found that newborn T4 and TSH values were considered normal, though demonstrated a wide range of values (T4: 2.7–42 µg/dl, and TSH: 3.0–114.0 ng/dl), perhaps reflecting the 48 h postpartum sampling range in this study (29). However, no prior work has reported on the relationship between thyroid function during pregnancy in alcohol dependent women and infant developmental outcomes.

Thyroid hormones are essential for normal healthy growth and neurodevelopment. The effect of subclinical hypothyroidism during the antenatal period on pregnancy outcomes has been a focus of attention (30–36). The largest study to date suggested an association of subclinical hypothyroidism with increased risk of premature delivery (37). Studies reporting on the effects of maternal thyroid hypofunction on fetal neurocognitive development have yielded inconsistent findings including one highly powered study showing no significant adverse effect on the intelligence quotient of 3- and 5-year-old children whose mothers had subclinical hypothyroidism in pregnancy (38, 39). In contrast, maternal subclinical hypothyroxinemia has overwhelmingly been reported as significantly associated with lower motor and intellectual development in children (40–53). While, a recent meta-analysis concluded that low maternal FT4 in pregnancy is associated with a threefold risk of delayed cognitive development in children (54), much remains to be learned about the relevant contributing mechanisms, particularly as it relates to maternal alcohol consumption. Our hypothesis is that alcohol consumption in pregnancy alters not only the maternal thyroid function, but that of the mother–fetus dyad. Thus, child neurodevelopment of alcohol consuming mothers may be affected even if maternal thyroid hormone levels are within the normal range.

Animal studies have provided some important insights into the relationships between alcohol exposure during pregnancy and thyroid function. Such studies have identified that placental levels of the thyroid hormone metabolizing enzyme, iodothyronine deiodinase 3 (Dio3), are increased following prenatal alcohol exposure (PAE) (14). Thus, with increased levels of placental Dio3, more T4 and T3 are metabolized into the biologically inactive reversed T3 and T2, respectively, leading to reduced amounts of T4 and T3 reaching the fetus. This is concerning, given the association between maternal FT4 levels and cognition of children at 3–5 years of age (52). The determining factor in thyroid hormone-dependent fetal brain development is the amount of the bioactive T3 in the brain, which is converted locally from T4. Since Dio3 expression is increased in the hippocampus of the PAE offspring, just as in the placenta, an additional decrease of hippocampal T3 is very likely, which is exaggerated by the reduced levels of T4 reaching the brain (16, 55, 56). These events can be precipitated by alcohol during development even in the face of adequate maternal peripheral T4 levels. Some of these mechanisms cannot be and others have not yet been explored in humans.

The current study investigated the impact of peripheral thyroid function of alcohol-consuming pregnant women on child developmental outcomes. We assessed serum FT4, FT3, and TSH levels in pregnant women with moderate to severe alcohol consumption and in those without significant alcohol consumption. We further assessed growth parameters at birth and neurodevelopmental outcomes of their children at 6 and 24 months of age.

## Materials and Methods

### Participants

The study was embedded in the Drakenstein Child Health Study (DCHS), a multi-disciplinary longitudinal birth cohort study investigating the early biological and psychosocial determinants of child health in two communities in the Western Cape Province, South Africa (57, 58). Both communities are largely low socioeconomic status and are characterized by a high prevalence of psychosocial risk factors. These include high rates of drug and alcohol usage (57), maternal psychological distress and depression (59), community exposure to violence and intimate partner violence (60), and low levels of employment and educational attainment (57). The population is stable, with little immigration or emigration. In excess of 90% of people in the district use the public-sector health system.

The methods of the larger DCHS are described elsewhere (57, 58). Briefly, the cohort consisted of a South African sample of English, Afrikaans, or isiXhosa speaking mother–infant dyads. Mothers were recruited into this population-based cohort study from two clinics between March 2012 and March 2015 while attending for antenatal appointments. Women were enrolled in the DCHS at 20–28 weeks’ gestation and were followed through birth and postnatally. Pregnant mothers were considered eligible for inclusion if they were 18 years or older, were attending antenatal care at one of the two clinics and planned to remain resident in the region for at least 1 year. Mothers who were eligible and agreed to be included, provided informed written consent, in their preferred language, at enrollment and were re-consented annually following childbirth. Informed consent forms described the scope and aims of the study, including potential harm or benefits. The study was approved by the faculty of Health Sciences, Human Research Ethics Committee, University of Cape Town (401/2009), Stellenbosch University (N12/02/0002), and the Western Cape Provincial Health Research committee (2011RP45).

In this pilot study, 21 mothers and their children with moderate–severe (PAE, as defined by mothers drinking at least twice a week and at least two drinks per occasion during any stage of pregnancy prior to recruitment) and 19 mothers and their children with no alcohol exposure (controls) were assessed for maternal thyroid function and child growth and developmental outcomes (61).

### Child Assessment

Children were assessed by formally measured growth parameters, and a subgroup had their development evaluated using the Bayley III Scales of Infant Development (BSID III) at 6 and 24 months of age (62). Assessors were blinded to alcohol and thyroid hormone status of the mothers. This assessment of infant development has been widely used globally and has been further validated in a South African population (63). The BSID-III consists of five subscales that were evaluated through direct observation by a trained assessor: cognitive, receptive language and expressive language, fine and gross motor. In addition, two additional scales were assessed through a caregiver questionnaire: Social-Emotional and Adaptive Behavior that can be used to indicate emotional and behavioral dysregulation. Scaled scores adjusted for age are reported, standardized mean 10, SD 3. Quality control was performed on all the BSID-III assessments. Infants were excluded if born <36 weeks or had a low Apgar score (<7 at 5 min) and/or admission for hypoxic ischemic encephalopathy or other significant neonatal complication (such as neonatal jaundice requiring phototherapy). Infants were also excluded if they had an identified genetic syndrome or congenital abnormality.

### Thyroid Function Measures

Maternal blood samples were collected in mid-pregnancy, in moderate to severe alcohol-consuming women (PAE) at 5.36 ± 0.92 months, and in abstinent women at 5.18 ± 1.14 months of pregnancy.

For the thyroid function measurements, electrochemiluminescence immunoassays “ECLIA” (Roche Diagnostics GmbH, D-68305 Mannheim) were used on the Elecsys e-immunoanalyzers. The serum TSH assay measures 0.005–100 μIU/mL TSH with 2–8% interassay coefficient of variation. The euthyroid values for non-pregnant test subjects are 0.270–4.20 μIU/mL, given by the manufacturer. The FT4 II assay can quantify 0.3–100 pmol/L FT4 with less than 4% interassay coefficient of variation. The euthyroid values for non-pregnant test subjects are given by the manufacturer as 12–22 pmol/L (2.5th and 97.5th percentile). Finally, the serum FT3 III assay range is 0.6–50 pmol/L FT3 with less than 3% interassay coefficient of variation. The euthyroid values for non-pregnant test subjects are given by the manufacturer as 3.1–6.8 pmol/L (2.5th and 97.5th percentile).

### Statistics

We generated descriptive statistics for maternal sociodemographic characteristics, infant anthropometric data, and BSID III Scaled Scores. The data were compared using Student’s *t*-tests for normally distributed continuous variables (e.g., maternal age), Pearson’s chi-square tests for categorical variables (e.g., ethnicity), and Mann–Whitney *U* tests for continuous variables that did not meet the assumptions for parametric testing (e.g., BSID Scaled Scores). Statistical analyses for this information was generated using SPSS (version 24).

**Figure 1:**
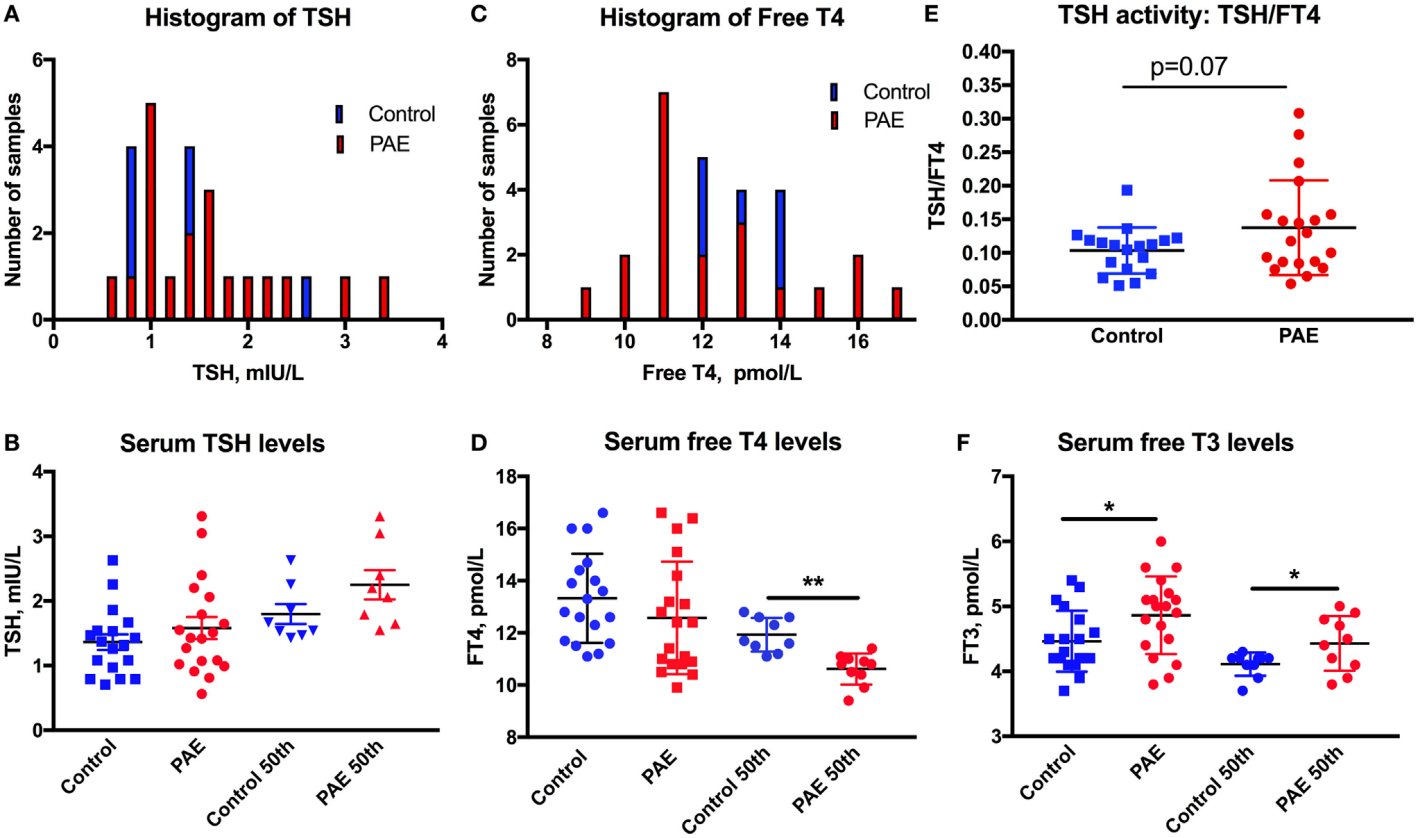
Thyroid function in pregnant women. The distribution is shifted toward higher levels of serum TSH **(A)**, toward lower levels of FT4 **(C)**, and greater ratio of TSH/FT4 **(E)** in pregnant women with moderate to severe alcohol consumption [prenatal alcohol exposure (PAE)] compared to no alcohol use women (Controls). Means and SEM of serum **(B)** TSH, **(D)** FT4, and **(F)** FT3 levels in PAE and control women, and the highest 50th percentile of TSH, lowest 50th percentile FT4 and FT3, respectively. *p < 0.05; **p < 0.01. Trending significance is shown by the actual *p*-value.

Serum hormone levels in PAE and abstinent cases were compared using Student’s *t*-test for the complete dataset or for the upper and lower 50th percentile in the case of serum TSH and FT4, respectively. Please note that Bonferroni correction was not applied in the descriptive statistics (Table 1) and in the maternal thyroid function data (Figure 1). A significant body of literature argues that while the Bonferroni correction directly targets the Type 1 error problem, it does so at the expense of Type 2 error. Therefore, many statisticians recommend reporting all of the individual *p* values and making it clear that no mathematical correction was made for multiple comparisons (64).

**Table 1:** Maternal demographic, infant anthropometric, and developmental characteristics.

	Alcohol exposed (*n* = 21)	Controls (*n* = 19)	Statistics
Maternal age (years, SD)	27.16 (6.01)	28.93 (5.80)	*t* = −0.95, *p* = 0.35
Ethnicity (black/colored)	18/3	11/8	*χ*^2^ = 3.87, *p* = *0.05*
Infant sex (male/female)	10/11	16/3	*χ*^2^ = 5.87, *p* = *0.02*
Gestational age (weeks, SD)	38.43 (2.87)	38.63 (1.61)	*t* = −0.27, *p* = 0.79
Birth weight (kg, SD)	2.69 (0.56)	3.12 (0.62)	*t* = −2.27, *p* = 0.03
Length (cm, SD)^a^	48.65 (3.33)	50.79 (3.22)	*t* = −2.04, *p* = 0.05
Head circumference (cm, SD)	32.33 (2.42)	34.04 (2.38)	*t* = H2.25, *p* = 0.03
**6m BSID scaled scores**			
Cognitive^b^	9.82 (2.23)	9.17 (2.12)	*U* = 49.00, *p* = 0.32
Receptive communication^b^	11.27 (2.49)	8.92 (2.31)	*U* = 37.00, *p* = 0.08
Expressive communication^b^	11.82 (2.36)	8.92 (3.68)	*U* = 32.00, *p* = 0.04
Fine motor^b^	12.09 (1.92)	11.33 (3.11)	*U* = 62.00, *p* = 0.83
Gross motor^b^	9.18 (3.68)	9.50 (1.62)	*U* = 65.00, *p* = 0.98
Socio emotional development^b^	10.82 (2.82)	10.42 (3.70)	*U* = 65.00, *p* = 0.98
**24m BSID scaled scores**			
Cognitive^c^	7.06 (2.01)	6.88 (1.71)	*U* = 116.00, *p* = 0.49
Receptive communication^d^	6.94 (1.61)	6.81 (1.97)	*U* = 127.00, *p* = 0.99
Expressive communication^e^	7.27 (1.87)	7.19 (1.87)	*U* = 112.00, *p* = 0.77
Fine motor^c^	8.76 (2.68)	9.81 (3.25)	*U* = 109.50, *p* = 0.34
Gross motor^f^	8.13 (2.90)	9.07 (2.66)	*U* = 83.50, *p* = 0.15
Socioemotional development^c^	13.76 (3.13)	12.50 (3.06)	*U* = 107.50, *p* = 0.31

*^a^Data missing for single alcohol exposed participant*.

*^b^Data missing for 10 alcohol exposed and 7 healthy unexposed (control) participants*.

*^c^Data missing for 4 alcohol exposed and 3 healthy unexposed (control) participants*.

*^d^Data missing for 5 alcohol exposed and 3 healthy unexposed (control) participants*.

*^e^Data missing for 6 alcohol exposed and 3 healthy unexposed (control) participants*.

*^f^Data missing for 5 alcohol exposed and 4 healthy unexposed (control) participants*.

Pearson correlations were used to assess associations between serum hormone levels and child developmental measurements. FDR-corrections were employed for multiple comparisons, significance was set at *p* < 0.05. Linear regression analyses were carried out for maternal thyroid function measures and child developmental measures. Statistical analyses were carried out by Graphpad (Prism 7.0).

## Results

Table 1 presents the demographic characteristics of the study participants. The two groups of mothers were well matched for age and reasonably matched for ethnicity. Performance on the BSID III in this small cohort did not show significant group differences, with the exception of expressive communication at 6 months of age, but this pilot study was not designed or adequately powered to address this particular question.

Thyroid function of the pregnant women was assessed by serum TSH, FT4, and FT3 measurements. One women from the PAE group and two from the abstinent group had serum TSH levels above 3.5 mIU/L (3.65, 3.57, and 5.76 mIU/L), and so were removed from the dataset. The histograms of serum TSH and FT4 (Figures 1A,C) show the propensity of TSH values to spread toward the higher, and FT4 levels toward the lower levels in the PAE pregnant women. TSH levels did not show significant differences between PAE and abstinent women (Figure 1B). However, while FT4 levels did not differ between the groups, the lower 50th percentile of FT4 values were significantly lower in the PAE compared to abstinent women [*t*(17) = 4.6, *p* < 0.01; Figure 1D]. Similarly, the TSH to FT4 ratio tended to be greater in the PAE group [*t*(36) = 1.86, *p* = 0.07; Figure 1E], consistent with an exaggeration in the inverse relationship of serum TSH and FT4 in the PAE group. In contrast to FT4, serum FT3 levels were significantly higher in the PAE group compared to abstinent pregnant women [whole group: *t*(36) = 2.30, *p* < 0.05; lowest 50th percentile: *t*(17) = 2.20, *p* < 0.05; Figure 1F]. The increased serum levels of FT3 and unchanged or decreased FT4 suggest enhanced T4 to T3 conversion in the periphery.

**Figure 2:**
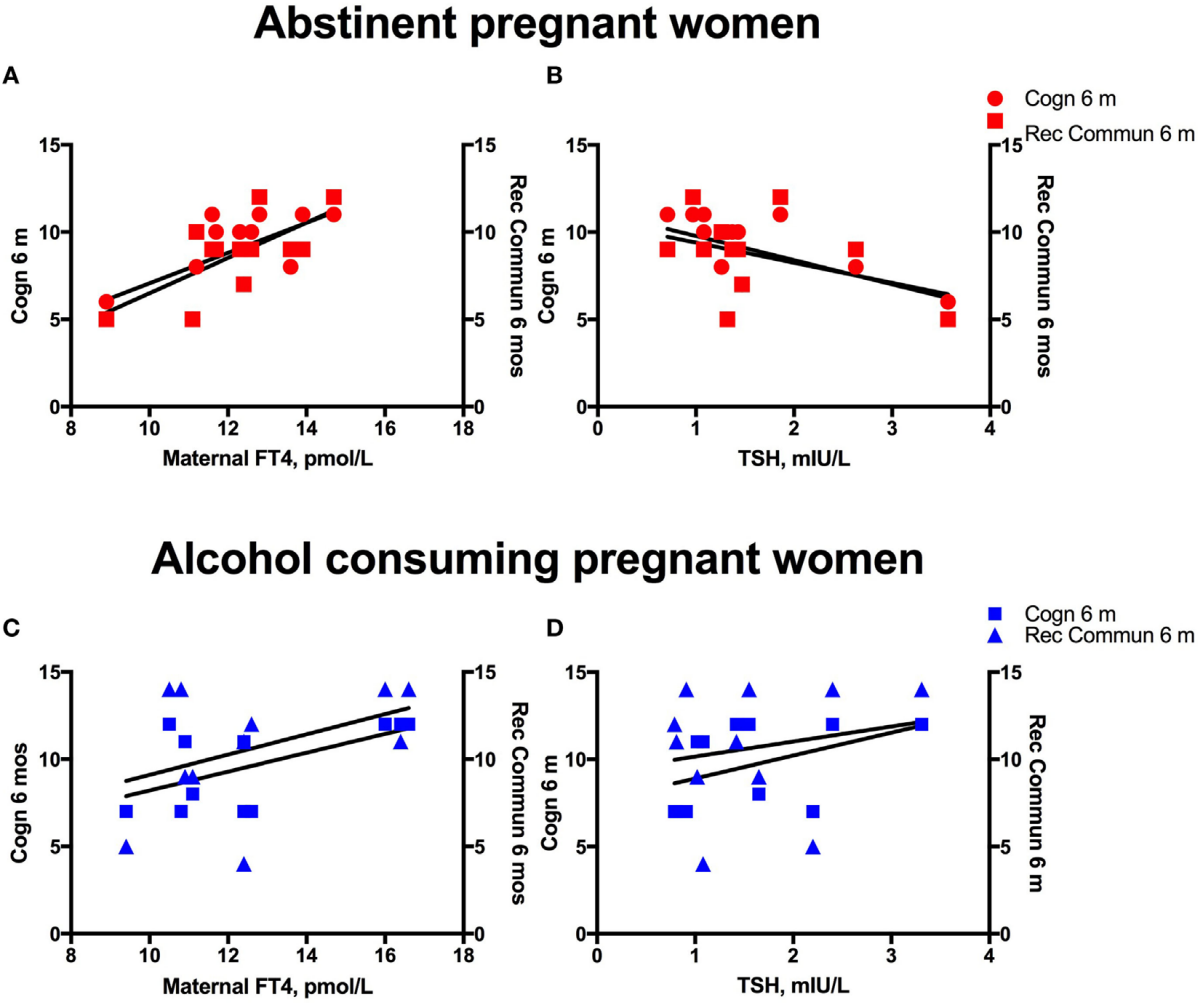
Alcohol consumption interferes with the predictive association between maternal thyroid measures and child development. Linear regression analyses between maternal serum FT4 and TSH, and cognitive, and language developmental measures of their children at 6 months of age in women abstinent during pregnancy **(A,B)** and pregnant women with moderate to severe alcohol consumption **(C,D)**.

We investigated the maternal thyroid function and infant developmental measures separately for abstinent women and infant dyad and for the alcohol-consuming mother infant dyad. As alcohol affects not only maternal thyroid function but also the developing fetus combining abstinent and alcohol-consuming mothers’ thyroid indices and their infants’ developmental measures may be misleading. In the abstinent women, correlation between maternal thyroid hormone indices during pregnancy and newborn and child measures found that increased maternal FT4 is significantly associated with greater scores on cognitive measures at 6 and 24 months of age and with increased performance in gross motor skills at 24 months of age (Table 2). In contrast, these relationships were not found in PAE dyads (Table 3), but in this group maternal FT3 levels significantly correlated with children’s social-emotional developmental scores at 24 months of age. Linear regression analyses between maternal FT4 and TSH, and cognitive, and language developmental measures of their children at 6 months of age are shown in Figure 2. Higher maternal FT4 levels in abstinent pregnant women predict greater cognitive and communicative functions of their children at 6 month of age [cognition 6 m: *F*(1, 10) = 6.08; *p* < 0.05; receptive communication 6 m: *F*(1, 10) = 9.09; *p* = 0.01; Figure 2A]. Maternal serum TSH shows the predicted inverse trend with these measures, albeit not significantly [cognition 6 m: *F*(1, 10) = 3.67; *p* = 0.08; receptive communication 6 m: *F*(1, 10) = 2.06; *p* = 0.18; Figure 2B]. Neither FT4 nor TSH of the pregnant alcohol-consuming women showed a significant relationship with these measures, which displayed a distinctly greater scatter than in the abstinent dyads as represented in Figures 2C,D.

**Table 2:** Correlation coefficients (Pearson) between thyroid function measures in abstinent pregnant women and their children’s birth and neurobehavioral parameters.

	1.	2.	3.	4.	5.	6.	7.	8.	9.	10.	11.	12	13.	14.	15.	16.	17.
1. Mat TSH																	
2. Mat fT4	0.231																
3. Mat fT3	−0.175	0.345															
4. Birth weight	−0.483	−0.242	0.390														
5. Birth length	−0.153	−0.103	−0.023	0.577*													
6. Head circum.	−0.209	−0.004	0.331	0.776*	0.287												
7. Cogn 6m	−0.518	0.615^#^	−0.016	0.123	0.341	0.218											
8. Rec Comm 6m	−0.413	0.690*	0.025	−0.064	0.307	0.019	0.825*										
9. Expr Comm 6m	−0.557	0.356	0.427	0.405	0.585	−0.023	0.123	0.363									
10. Fine motor 6m	−0.571	0.200	0.212	0.189	0.489	−0.148	0.262	0.450	0.700*								
11. Gross motor 6m	−0.014	−0.393	−0.014	0.237	0.458	0.098	0.000	0.077	0.221	0.703*							
12. Soc Emot Dev 6m	−0.349	0.232	0.122	0.134	0.366	−0.068	0.356	0.215	0.184	0.473	0.309						
13. Cogn 24m	−0.032	−0.231	0.029	0.402	0.492	0.483	0.227	0.354	−0.011	−0.154	−0.018	−0.178					
14. Rec Comm 24m	−0.259	0.382	0.433^#^	0.167	0.011	0.319	0.603^#^	0.713*	0.038	0.076	−0.074	0.014	0.391				
15. Expr Comm 24m	0.442	0.582*	−0.048	−0.556*	−0.503	−0.245	0.032	0.298	0.022	−0.231	−0.368	−0.215	−0.117	0.398			
16. Fine motor 24m	−0.095	0.110	−0.015	0.367	0.416	0.321	0.363	0.416	0.257	0.239	0.136	0.079	0.560*	0.545^#^	0.218		
17. Gross motor 24m	−0.034	0.514^#^	0.112	0.090	−0.126	0.361	0.563	0.520	0.011	−0.266	−0.293	−0.472	0.049	0.604*	0.522^#^	0.422	
18. Soc Emot Dev 24m	−0.072	0.101	0.345	0.148	0.168	0.182	0.128	0.123	0.287	0.247	0.232	−0.076	0.178	0.313	0.151	0.116	–0.032

FDR corrected p-values *p < 0.02; ^#^p < 0.04 for significant and trending, respectively.

**Table 3:** Correlation coefficients (Pearson) between thyroid function measures in the moderate to severe alcohol consuming pregnant women and their children’s birth and neurobehavioral parameters.

	1.	2.	3.	4.	5.	6.	7.	8.	9.	10.	11.	12.	13.	14.	15.	16.	17.
1. Mat TSH																	
2. Matf T4	0.225																
3. Matf T3	−0.136	0.327															
4. Birth weight	−0.326	0.366	0.348														
5. Birth length	−0.015	0.277	0.354	0.740*													
6. Head Circum	−0.323	0.116	0.141	0.838*	0.523*												
7. Cogn 6m	0.437	0.578	0.392	0.603	0.488	0.404											
8. Rec Comm 6m	0.190	0.411	0.468	0.418	0.386	0.491	0.205										
9. Expr Comm 6m	0.095	0.504	0.322	0.711*	0.758*	0.477	0.722*	0.256									
10. Fine motor 6m	0.364	0.546	0.433	0.722*	0.804*	0.641^#^	0.793*	0.485	0.887*								
11. Gross motor 6m	−0.160	0.321	0.408	0.448	0.446	0.559	0.086	−0.010	0.538	0.442							
12. Soc Emot Dev 6m	0.104	−0.351	−0.120	−0.218	0.249	−0.026	−0.282	0.100	−0.039	0.166	0.247						
13. Cogn 24m	−0.294	0.062	0.460	0.383	0.303	0.131	−0.056	0.459	0.452	0.397	0.662	0.203					
14. Rec Comm 24m	−0.419	0.416	0.512	0.488	0.558^#^	0.316	0.188	0.531	0.648	0.587	0.700^#^	−0.072	0.677*				
15. Expr Comm 24m	−0.084	0.244	0.446	0.586^#^	0.547^#^	0.174	0.290	0.422	0.613	0.571	0.466	−0.221	0.754*	0.797*			
16. Fine motor 24m	−0.134	0.095	0.232	0.415	0.201	0.245	0.513	−0.456	0.728^#^	0.402	0.590	−0.399	0.241	0.325	0.327		
17. Gross motor 24m	−0.176	−0.176	0.418	0.072	−0.105	0.268	−0.075	0.303	−0.099	−0.023	0.280	−0.136	0.483	0.328	0.323	0.174	
18. Soc Emot Dev 24m	−0.015	0.280	0.569*	0.369	0.259	0.477	0.465	0.579	0.428	0.725^#^	0.574	0.259	0.233	0.514	0.226	0.352	0.436

FDR corrected p-values *p < 0.02; ^#^p < 0.04 for significant and trending, respectively. Red: gained significance compared to control group, blue: lost significance compared to control group.

## Discussion

This pilot study suggests that moderate-to-severe alcohol consumption during pregnancy is associated with alterations in maternal thyroid function, particularly with increased serum FT3 levels. Increased serum FT4 levels in abstinent pregnant women are significantly associated with higher scores on cognitive measures in early childhood in their children. In contrast, in women with moderate-to-severe alcohol consumption, this association is not present, but instead, increased serum FT3 levels are significantly associated with altered social-emotional development at 24 months of age.

Altered thyroid function in pregnant women who consumed alcohol was reflected in increased FT3. Furthermore, serum TSH tended to be higher, and FT4 tended to be lower in PAE women. Increased serum FT3 in alcohol consuming pregnant women is a novel and unexpected finding. In the light of the tendency of alcohol lowering FT4, increased serum FT3 levels could be due to greater proportion of T4 converted to T3 by increased expression of deiodinase 2 (*Dio2*) after alcohol exposure. Chronic alcohol exposure is known to increase deiodinase 1 in the liver (65) and deiodinase 2 in the pituitary and the frontal cortex (66) as well as the amygdala (67). Furthermore, in a positive feedforward regulation, T3 is known to induce *Dio2* expression in brain regions (68) and in the brown adipose tissue as well (69). An example of how increased Dio2 can coexist with decreased T4 and increased T3, at least in the brain, is shown by the effects of a single dose lithium (67).

Importantly, with greater T4 to T3 conversion in the periphery, less T4 is available to cross to the placenta to the fetal brain. Since only a portion of brain T3 originates from the periphery and the main source is local conversion from T4, the potentially reduced amount of T4 reaching the fetal brain can induce a state of hypothyroidism in the fetal brain. Furthermore, some thyroid hormone transporters prefer T4 to T3; therefore, brain region-specific hypothyroidism can occur in the presence of decreased serum FT4 and elevated FT3 (70). Interestingly, decreased serum FT4 and increased FT3 has been described in patients with posttraumatic stress disorder (PTSD) (71) and in women at risk for postpartum depression (72). Whether that finding is related to alteration in cortisol levels in subjects with PTSD (73), which is also known to occur in alcohol consuming pregnant animals (74, 75) is not known. Additionally, elevated FT3 has been shown to correlate with increases in preterm birth (76), which is very frequent in PAE pregnancies.

The finding here of a correlation between FT4 and cognitive outcomes is consistent with much work showing the reverse relationship: adverse outcomes in children born to mothers with isolated hypothyroxinemia (30, 35, 38, 40, 41, 50, 77–90). Adverse outcomes of hypothyroxinemia reported in populations across the world, include prematurity, lower IQ, general cognitive developmental deficit, language delay, impaired motor function, smaller head circumference among others. In the present study, serum FT4 levels of abstinent women in pregnancy were linearly correlated with their children’s cognitive performance in infancy in the predicted direction. However, this seemingly direct linear relationship between euthyroxinemic maternal milieu and child cognitive development was abolished by alcohol consumption during pregnancy in our pilot cohort. The possibility that an increased proportion of FT4 converted to FT3 in the PAE women suggests potentially decreased FT4 crossing into the placenta. Should placental levels of DIO3 be increased after PAE in humans, as it has been shown in animal studies, even less FT4 and FT3 would reach the fetus. Thus, even in the context of non-significant differences in FT4, in the presence of PAE during this important developmental window, the placental transfer of FT4 and fetal thyroid hormone utilization may differ. Whether subclinical hypothyroidism or hypothyroxinaemia during pregnancy is an additional risk factor for alcohol-induced neurodevelopmental disorder is important to explore. Unfortunately, there remains great controversy about the significance of subclinical hypothyroidism in general, but in pregnancy, in particular. Should alcohol consumption in pregnancy alter not only the maternal thyroid function but that of the mother–fetus dyad, then neurodevelopment of the children alcohol consuming mothers may be affected even in the presence of maternal thyroid hormone levels within the normal range. This is the proposed potential mechanism (at least in part) for the effects of alcohol exposure on the developing fetus, and a potential pathway for treatment.

The finding of linear association between the elevated serum FT3 levels in the alcohol consuming pregnant women and their infant’s social and emotional development is difficult to interpret. Without detailed interpretation of this developmental measurement, one can venture the opinion that this relationship may cover multiple aspects of emotional development. Specifically, very young children with FASD have been described as being “excessively friendly and fearless” (91), “friendly and trusting with strangers” (92), thus clearly indicating differences in their emotional, social development from the control population. This verbal, but impulsive, disposition may be the signature that is being picked up in this domain (93). The actual items measured in this domain at this age include requesting physical and social need through verbal or visual means. Thus, a more demanding or forceful child may score highly on these particular items without socio-emotional flexibility, which is one of the hallmark deficits of the FASD profile. As with all early developmental outcome studies, the answers may only manifest with larger samples.

A number of limitations to this pilot study should be emphasized. In particular, the small sample size, the lack of maternal thyroid indices at earlier time points in pregnancy and additional measurements of peripheral thyroid function, resulting in the conservative interpretation of the findings. Nevertheless, these are the first data to report child developmental outcomes in relation to maternal thyroid indices in the context of PAE. These findings are supported by animal work where thyroid hormone efficacy and metabolism is affected by alcohol exposure during pregnancy with an impact on the offspring cognitive outcomes compared to those infants born to non-alcohol consuming mothers (55, 56). Future studies, in addition to expanding the sample size, will investigate changes in maternal and child thyroid hormone profile in the context of PAE and in healthy unexposed pregnancies over the course of pregnancy. Additionally, longitudinal developmental trajectories into later childhood should be used to investigate the mediating role of thyroid function in pregnancy on child development in future studies involving abstinent and moderate to severe alcohol consuming women.

## Ethics Statement

The study was approved by the faculty of Health Sciences, Human Research Ethics Committee, University of Cape Town (401/2009), Stellenbosch University (N12/02/0002), and the Western Cape Provincial Health Research committee (2011RP45). All subjects gave written informed consent in accordance with the Declaration of Helsinki.

## Author Contributions

ER, DS, and KD conceived the research question, refined the approach for this specific project. ER performed the analysis. KD and ER provided the interpretation of the results and jointly wrote the first draft of the manuscript. HZ conceived and is lead investigator of the umbrella birth cohort and DS the psychosocial arm in which this pilot analysis was embedded and as such facilitated the data collection and study design. WB, NH, and CW were all involved in data collection, quality control, and interpretation of both maternal and infant outcome measures in specific content areas. All authors reviewed and approved the final draft of this manuscript.

## Conflict of Interest Statement

The authors declare that the research was conducted in the absence of any commercial or financial relationships that could be construed as a potential conflict of interest.
